# Increased Incidence of Thyroid Dysfunction and Autoimmunity in Patients with Vernal Keratoconjunctivitis

**DOI:** 10.1155/2014/804870

**Published:** 2014-07-20

**Authors:** Stefano Stagi, Neri Pucci, Laura Di Grande, Cinzia de Libero, Roberto Caputo, Stefano Pantano, Ivan Mattei, Francesca Mori, Maurizio de Martino, Elio Novembre

**Affiliations:** ^1^Department of Health Sciences, University of Florence, Anna Meyer Children's University Hospital, Viale Pieraccini 24, 50139 Florence, Italy; ^2^Paediatric Allergology Unit, Anna Meyer Children's University Hospital, Viale Pieraccini 24, 50139 Florence, Italy; ^3^Clinical Ophthalmology Unit, Anna Meyer Children's University Hospital, Viale Pieraccini 24, 50139 Florence, Italy

## Abstract

Hormones may play a role in the pathophysiology of vernal keratoconjunctivitis (VKC). An increased incidence of thyroid autoantibodies was recently observed in VKC, although there were no data on thyroid function. Two hundred and eighty-eight patients (202 males, 86 females; range 5.5 to 16.9 years) with VKC were evaluated and compared with 188 normal age- and sex-matched subjects. In all subjects, serum concentrations of free T4, TSH, thyroperoxidase, thyroglobulin, and TSHr autoantibodies were evaluated. In VKC, the family history of thyroid diseases showed no significant differences compared to the controls (9.4 versus 8.6%), whereas the family history of autoimmune diseases was significantly higher (13.2% versus 6.3%; *P*<0.05). Subclinical hypothyroidism was diagnosed in 6.6% (versus 1.6% of the controls; *P*<0.05) and overt hypothyroidism in 0.7% (versus 0.0% of the controls; *P* = NS). Finally, 5.2% of patients were positive for thyroid autoantibodies, which were significantly higher with respect to the controls (0.5%, *P*<0.05). In the patients positive for thyroid autoantibodies, 80% showed a sonography pattern that suggested autoimmune thyroiditis. Thyroid function and autoimmunity abnormalities are frequently present in children with VKC. Children with VKC should be screened for thyroid function and evaluated for thyroid autoimmunity.

## 1. Introduction

Vernal keratoconjunctivitis (VKC) is a severe inflammatory disease that commonly manifests seasonally during childhood, whereas a perennial and persistent form of the disease is less frequent [1].

The aetiology and immunopathogenesis of VKC remain unclear [1]; despite the assumption that VKC is part of the family of allergic diseases, a large proportion of the patients diagnosed have no familial or personal history of atopic disease and can have negative results on the standard allergic diagnostic tests. However, antiallergic therapy is often unsuccessful [1]. In VKC patients, the male-female ratio has been reported to range from 4 : 1 to 2 : 1 [2].

All types of VKC are characterised by intense itching, tearing, mucous secretions, and severe photophobia. Cytological, biohumoral, immunohistological, and molecular biological studies indicate that VKC is a Th2 lymphocyte-mediated disease. Mast cells and eosinophils, as well as their mediators, play major roles in the clinical manifestation of VKC. In addition to typical Th2-derived cytokines (IL-4, IL-5, and IL-13), other cytokines, such as chemokines, growth factors, and enzymes, are overexpressed in the conjunctiva of VKC patients [2, 3].

Because of the notable difference between genders and the resolution of this disease after puberty, it has been suggested that hormonal factors play a role in the development of VKC by influencing the immune deviation and the response to environmental factors, in addition to genetic, unknown, and predisposing conditions [2].

Sex hormones may also play a relevant role in the pathophysiology of allergic diseases by reciprocal interactions between the immune and endocrine systems. Oestrogen and progesterone have been shown to be active players in the ocular immune system. In an immunohistochemical study of patients with VKC, oestrogen and progesterone receptors were shown to be overexpressed in eosinophils and other inflammatory cells on the conjunctiva [2]. These hormones may bind to conjunctival receptors and exert a proinflammatory effect through the recruitment of eosinophils to the conjunctival tissue [4].

Sex hormone-related diseases, such as gynaecomastia, polycystic ovary syndrome, mammary fibroadenoma, and adiposogenital dystrophy, or autoimmune diseases have been reported in 2% of patients suffering from VKC [5]. However, recent data reported that VKC patients may have a higher frequency of a family history of autoimmune disorders [6], thyroid autoantibodies [7], and antinuclear antibodies (ANA) [6, 8]. Thus, the purpose of this study was to evaluate the prevalence of abnormalities of thyroid function and autoimmunity in a cohort of patients with VKC.

## 2. Subjects and Methods

Two hundred and eighty-eight consecutive patients (202 males, 86 females; median age 9.7, range 5.5 to 16.9 years) with VKC were recruited from July 2005 to February 2012 at the Paediatric Allergology Unit at Anna Meyer Children's University Hospital in Florence, Italy.

Ethical approval was obtained from the Ethics Committee at Anna Meyer Children's University Hospital. After an explanation of the nature of the study, written informed consent was obtained from the parents.

### 2.1. Case Definition and Study Protocol

**Figure 1:**
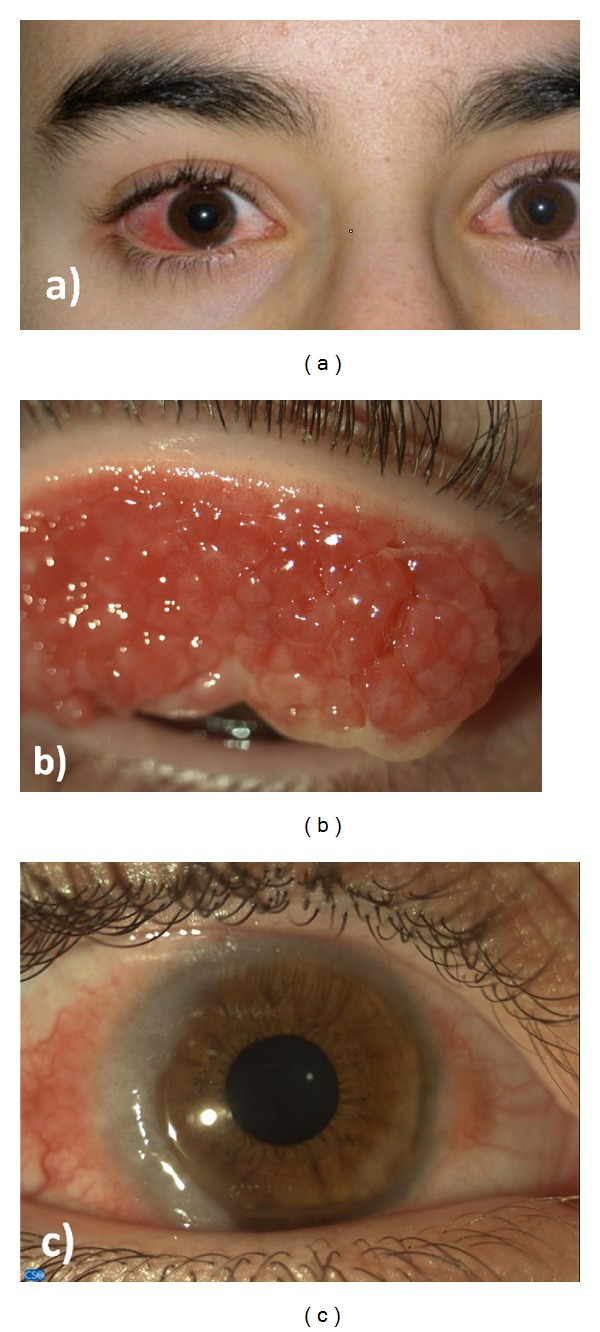
Severe conjunctival redness in a male with vernal keratoconjunctivitis (a); “cobblestones” giant papillae on the upper tarsal conjunctiva (b); Horner-Trantas dots (raised, white accumulations of eosinophils), associated with gelatinous thickened and opacification of the limbus with copious amounts of mucoid discharge (c).

A diagnosis of VKC was established according to the presence of giant tarsal papillae and/or limbal papillae relapsing in the spring and summer [9]. The symptoms were associated with a mild to severe cobblestone-like appearance in the upper tarsal conjunctiva, mucous discharge, epithelial keratopathy, and at least two eosinophils per optic field (100x, oil immersion lens) in the conjunctival scraping. Finally, each patient was evaluated to distinguish between the limbal, tarsal, and mixed forms, as well as between seasonal and perennial symptoms (Table 1 and Figure 1).

The skin prick test, specific IgE serum concentrations, and peripheral blood eosinophil count were also performed [1].

Parents were asked when their children first manifested symptoms of VKC. The possible prolonged administration of either systemic or topical drugs, including antihistamines, corticosteroids, cyclosporine, and nonsteroidal anti-inflammatory drugs, was also investigated.

In all of the subjects, the serum concentrations of free-T_3_ (FT_3_), free-T_4_ (FT_4_), thyroid stimulating hormone (TSH), thyroperoxidase autoantibodies (TPOA), thyroglobulin autoantibodies (TgA), and TSH-receptor (TSHr) autoantibodies, as well as ultrasonographic data when appropriate, were evaluated. Additionally, continuous data such as height, pubertal staging, weight, and body mass index (BMI) were collected; the last parameter was considered as the standard deviation score (SDS).

Finally, all of the patients and control subjects and/or their parents were interviewed about their family history of autoimmune diseases up to their second-degree relatives. The following autoimmune diseases were considered: autoimmune thyroid diseases, rheumatoid arthritis and other rheumatological disorders, coeliac disease, type 1 diabetes mellitus, vitiligo, alopecia, multiple sclerosis, and inflammatory bowel disease [10].

Exclusion criteria from the study for VKC patients and controls were the presence of a history of an already diagnosed thyroid or autoimmune disease.

### 2.2. Thyroid Function

FT_4_, FT_3_, and TSH serum levels were measured using immunometric assays (Immulite 2000, Third Generation, DPC Diagnostic Products Corporation, Los Angeles, CA, USA). Interrun coefficients of variation were <8.5% for TSH, <7.5% for FT_4_, and <9.1% for FT_3_. The normal ranges were adapted by Stagi et al. as previously reported [11].

Subclinical hypothyroidism was defined as a TSH level above the upper reference limit for age in conjunction with normal serum thyroid hormone levels. Overt hypothyroidism was defined as persistently increased TSH levels with decreased serum thyroid hormone levels.

### 2.3. Thyroid Antibodies

Thyroid autoimmunity was evaluated using fluorescence enzymatic immunoassays of TgA and TPOA antibodies; positive TgA values were considered to be ≥50 IU/mL and TPOA ≥ 100 IU/mL [10]. TSHr autoantibodies were measured with THBIA (DiaSorin Spa, Vercelli, Italy) using a two-step radioreceptor assay; TSHr was considered to be positive when values were >9 U/L.

### 2.4. Thyroid Sonography

The thyroid volume and morphology, when appropriate, were investigated using an Advanced Technology Laboratories (ATL) high-definition imaging (HDI) 5000 ultrasound scanner (ATL Ultrasound, Bothell, WA, USA) in combination with a 5–12 MHz linear transducer. Thyroid sonography was performed by experienced radiologists who were unaware of the diagnosis, thyroid hormones, or antibody levels of the subjects.

### 2.5. Control Group

To compare thyroid function and autoimmunity, a group of 188 (median age 9.5, range 1.8 to 14.6 years, *P* = NS) normal age- and sex-matched subjects with no history of atopic ocular/systemic diseases and who have been admitted to the hospital for minor surgery (e.g., adenotonsillectomy, phimosis, dermoid cyst, and herniotomy) were selected from the same geographical area. Studies regarding a portion of this group have been previously reported [10, 11].

## 3. Statistical Analysis

Statistical analyses were performed using SPSSX (SPSSX Inc., Chicago, IL, USA). The *χ* ^2^-test or Fisher's exact test, when appropriate, was used to compare differences between the patients and control subjects. Bonferroni's correction for multiple comparisons was also applied in selected instances. Summaries of the continuous variables are given as the mean ± SD and the range. Statistical tests were two-tailed and considered to be significant when *P* < 0.05.

## 4. Results

The primary characteristics of thyroid function and autoimmunity in our cohort of VKC patients and controls are summarised in Table 1.

Associated allergic manifestations such as asthma, eczema, or rhinitis were observed in 34.9% of the patients, and a family history positive for allergic diseases was recorded in 29.5% of the VKC patients. A positive skin prick test for common allergens was observed in 52.1% of the patients, and serum containing specific IgE was detected in 53.7% of the patients.

Twenty-seven (9.4%) patients had a positive family history of thyroid disease (15 autoimmune thyroid diseases, 8 thyroid nodules, and 4 goitre), which was not significantly different compared to the control group (8.6%; *P* = NS). However, 38 (13.2%) of the VKC cases had a positive family history of autoimmune diseases, which was significantly higher compared to the control subjects (12 subjects, 6.3%, *P* < 0.05). Of these, 15 (5.2%) patients had a positive family history of autoimmune thyroid diseases (versus 3.7% of the control subjects, *P* = NS): 12 (4.2%) for Hashimoto's thyroiditis and 3 (1.0%) for autoimmune hyperthyroidism.

Subclinical hypothyroidism was diagnosed in 19 patients (6.6%) and overt hypothyroidism in 2 patients (0.7%). The frequency of subclinical hypothyroidism was significantly higher than the control group (3 patients, 1.6%, *P* < 0.05), whereas the frequency of overt hypothyroidism did not differ from the control group (0 subjects, *P* = NS).

**Figure 2:**
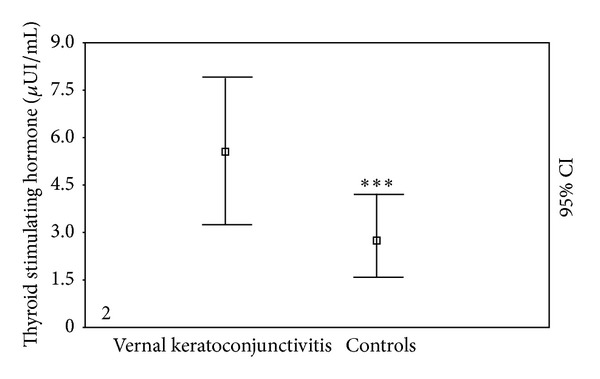
Thyroid stimulating hormone (TSH) levels in VKC patients compared to the control group. ***P < 0.001.

The FT_3_ levels were similar between the VKC patients (6.84 ± 0.66 pmol/L) and control subjects (6.51 ± 0.79 pmol/L, *P* = NS). The FT_4_ levels were also similar between the VKC patients and control subjects (15.52 ± 3.57 versus 15.13 ± 4.31 pmol/L, *P* = NS). However, the TSH levels were considerably higher in VKC patients compared to the control group (5.42 ± 2.31 versus 2.81 ± 1.24 *μ*UI/mL, *P* < 0.0001) (Figure 2); if patients with subclinical and overt hypothyroidism were excluded, the TSH levels were not significantly different compared to the control group (2.99 ± 0.97 versus 2.81 ± 1.24 *μ*UI/mL, *P* = NS).

Fifteen (5.2%) patients were positive for thyroid autoantibodies, and this percentage was significantly higher compared to the control group (1 subject, 0.5%, *P* < 0.05). Two patients with overt hypothyroidism were a part of this group. No patients were positive for TSHr autoantibodies. In the patients who were positive for thyroid autoantibodies, 12/15 (80%) showed a heterogeneous echotexture and diffuse hypoechogenicity on their thyroid sonography, whereas the remaining 3 patients showed normal echostructure and echogenicity (0.5%, *P* < 0.05). These 3 patients were determined to have subclinical hypothyroidism.

L-Thyroxine treatment became necessary for one patient with autoimmune thyroiditis and overt hypothyroidism because of the persistently increased TSH levels in this patient, whereas another patient was continually followed over time.

Upon evaluation of the different forms of VKC, we did not find differences regarding a family history of autoimmune diseases and thyroid diseases or of thyroid autoimmunity and function.

## 5. Discussion

Our data show, to the best of our knowledge for the first time, an increased prevalence of thyroid function abnormalities in VKC patients. However, our study confirms that patients with VKC also present a higher prevalence of thyroid autoimmunity, confirming the findings of Tesse et al. [7], who reported a higher rate of positivity of thyroid autoantibodies in patients with VKC compared to healthy subjects, despite the fact that thyroid function in the VKC patients was not studied. The same study also discovered that, in 181 VKC children, 22% were positive for AbTG, 14.6% for AbTPO, and 15.8% for ANA.

In evaluating thyroid function and thyroid sonography in VKC, our study suggests that these patients may be at a higher risk to develop autoimmune thyroiditis compared to the general population. These data are particularly important because a high percentage of VKC patients with thyroid autoantibodies also showed a higher rate of thyroid dysfunction, primarily manifesting as subclinical hypothyroidism. We observed that 33.3% of our patients with thyroid autoantibodies also presented with either subclinical or overt hypothyroidism, which emphasises the importance of evaluating thyroid function in VKC patients.

Our study showed that VKC patients have a significantly higher prevalence of a family history of autoimmune diseases, confirming the data of Zicari et al. [6] and Tesse et al. [7]. For example, Tesse et al. showed that 12.2% of VKC cases had a positive family history of psoriasis; 5.2% of the patient group was positive for thyroiditis, which suggests the role of common components associated with these immune-based diseases in the development of thyroid autoimmunity [7].

VKC has been associated with sex hormone-related diseases. Disorders such as gynaecomastia, polycystic ovary syndrome, mammary fibroadenoma, and adiposogenital dystrophy, as well as autoimmune diseases, have been reported by 2% of patients diagnosed with VKC [5].

It is also interesting to note that an excessive growth of eyelashes has been reported in patients with VKC [12], and this condition is associated with hypothyroidism in some genetic diseases [13].

The cause of the observed higher frequency of autoimmune thyroiditis and thyroid dysfunction in VKC patients is unclear. It is also unknown if VKC is associated with other autoimmune disorders. Further studies should be conducted to elucidate the possible mechanism behind this relationship. The significant increase in the frequency of a family history of autoimmune diseases disclosed by VKC patients may also suggest a role of common components of the immune system in the pathogenesis of these disorders.

Autoimmune thyroid diseases (AITD), which affect approximately 5% of the adult population (with a larger incidence in women), result from the disruption of self-tolerance induced by environmental factors in genetically susceptible individuals [14] and are characterised by lymphocytic infiltration of the thyroid, the generation of antibodies against thyroid antigens, and thyroid dysfunction. The clinical spectrum of autoimmune diseases encompasses Hashimoto thyroiditis (the most common form), Graves' disease, postpartum thyroiditis, drug induced thyroiditis, thyroiditis within polyglandular autoimmune syndromes, and atrophic thyroiditis [15].

Phenotypic expression of thyroid autoimmunity is dependent on the pattern of the immune response that predominates at any given time. Abnormal interactions between thyrocytes and immunocompetent (e.g., macrophages, dendritic cells, and T-cells) and other immune cells generate autoimmunity. However, Th2 immune responses also downregulate the Th1 immune response [16]. Because these subpopulations tend to function antagonistically toward one another, the balance between Th1 and Th2 lymphocytes may determine the outcome of autoimmune diseases [17].

Hashimoto thyroiditis is a typical T-cell-mediated autoimmune disease and is characterised by formation of tertiary lymphoid follicles within the thyroid (containing T-cells, increased Th1, and B cells), with the destruction of thyroid follicular cells generating hypofunction and the presence of anti-TPO and/or anti-Tg antibodies in the serum.

However, in patients with VKC, the clear abundance of Th2 cytokines, the upregulated expression of their receptors, and the conspicuous paucity of T helper cell type 1 (Th1) cytokines in the tears and serum confirm the crucial role of these factors in the onset and perpetuation of the chronic allergic inflammation observed in these patients [18]. The immune, nervous, and endocrine systems appear to interact with each other in the pathogenesis of VKC [19].

Although allergic diseases (Th2 disorders) and autoimmune diseases (Th1-mediated) are usually considered to be diametrically opposed in the immune response, this contention is now less evident because it has been found that allergy-associated mechanisms can contribute to the pathogenesis of autoimmune diseases, such as multiple sclerosis [20]. However, an association between the presence of wheezing as a measure of asthma and the occurrence of type I diabetes [21] as well as between an allergic constitution (asthma, atopic eczema) and AITD [22] was observed. Furthermore, there is a correlation between the elevated levels of immunoglobulin E and a slower decrease in TSH-receptor autoantibody levels, along with a lower chance of remission of the disease after antithyroid drug treatment in patients with Graves' disease [23, 24]. Additionally, patients with a relapse of Graves' hyperthyroidism had a higher rate of allergic rhinitis attacks compared to patients who went into remission [25].

It is interesting to note that there is also an association between another allergic disease (chronic urticaria) and Hashimoto's thyroiditis, as TPO and/or Tg autoantibodies were found more frequently in patients with chronic urticaria and angioedema compared to healthy controls [26].

## 6. Conclusions

Abnormalities of thyroid function and thyroid autoimmunity are present in children with VKC. The possible relationship between these disorders should be confirmed and further studied. Children with VKC should also be screened for thyroid function to evaluate possible thyroid autoimmunity in the presence of hypothyroidism.

## Figures and Tables

**Table 1:** Demographic data, familial occurrence of autoimmune and thyroid diseases, and prevalence of thyroid dysfunction and autoimmunity in vernal keratoconjunctivitis (VKC) patients and controls.

	VKC	Controls	*P*
Subjects (*n*)	288	188	—
Tarsal	23%	—	—
Mixed	16%	—	—
Limbal	61%	—	—
Males : females	202/86	133/55	NS
Median age (yr)	9.7	9.5	NS
Skin prick tests positivity (%)			
Dermatophagoides	72%	—	—
Gramineae	56%	—	—
Cat fur	12%	—	—
Familial occurrence of autoimmune diseases	13.2%	6.3%	<0.05
Familial occurrence of thyroid diseases	9.4%	8.6%	NS
FT_3 _(pmol/L)	6.84 ± 0.66	6.51 ± 0.79	NS
FT_4 _(pmol/L)	15.52 ± 3.57	15.13 ± 4.31	NS
TSH (*μ*UI/mL)	5.42 ± 2.31	2.81 ± 1.24	<0.0001
Subclinical hypothyroidism	6.6%	1.6%	<0.05
Overt hypothyroidism	0.7%	0.0%	NS
Autoimmune thyroiditis	5.2%	0.5%	<0.05

NS = not significant.
